# Benefits of Long-Term Continuation of Low-Dose Methimazole Therapy in the Prevention of Recurrent Hyperthyroidism in Graves' Hyperthyroid Patients: A Randomized Prospective Controlled Study

**DOI:** 10.1155/2022/1705740

**Published:** 2022-10-11

**Authors:** Raweewan Lertwattanarak, Tada Kunavisarut, Sutin Sriussadaporn

**Affiliations:** Division of Endocrinology and Metabolism, Department of Medicine, Faculty of Medicine Siriraj Hospital, Mahidol University, Bangkok, Thailand

## Abstract

**Background:**

The long-term continuation of the low-dose antithyroid drug (ATD) beyond the standard duration of ATD therapy of 12–18 months to prevent recurrent hyperthyroidism (RH) is recommended with low quality of evidence.

**Objectives:**

To examine whether long-term continuation of low-dose ATD beyond the recommended duration of treatment would provide a benefit in the prevention of RH in patients with Graves' hyperthyroidism (GH) who achieved euthyroid status with a standard course of ATD therapy.

**Methods:**

A 36-month prospective randomized controlled study was conducted in 184 patients who had first diagnosed GH and were treated with a standard regimen of ATD therapy using methimazole (MMI) until achieving euthyroidism that was stably maintained for at least 6 months with a low-dose of (2.5–5 mg/day) MMI. All patients had neither a history of adverse effects from MMI, recurrent GH, severe and active ophthalmopathy nor conditions known to affect thyroid function before randomization. The patients were randomized into 2 groups: one group (92 cases) was assigned to discontinue (DISCONT-MMI) and the other (92 cases) was assigned to continue low-dose MMI (CONT-MMI) that was taken at the time of enrollment. The patients in both groups were followed up at 3, 6, 12, 18, 24, 30, and 36 months. The rate of RH was compared between both groups, and the adverse effects and risk factors of RH were also studied.

**Results:**

At the end of the 36-month study, 83 cases in CONT-MMI and 90 cases in DISCONT-MMI were eligible for analysis. The cumulative rates of RH in CONT-MMI were significantly lower than those in DISCONT-MMI at every follow-up time point (1.2% *vs.* 11.2%, 6.8% *vs.* 18.4%, 11.0% *vs.* 27.2%, 11.0% *vs.* 35.0%, and 11.0% *vs.* 41.2% at 6, 12, 18, 24, and 36 months, respectively; *p* < 0.01). Cox proportional hazard multivariate analysis showed that there were 2 factors independently associated with the risk of RH, including continuation of low-dose MMI therapy, which decreased the risk of RH by 3.8 times (HR = 0.26, *p* = 0.007, 95% CI = 0.10–0.70) and age onset of hyperthyroidism before 40 years, which increased the risk of RH by 2.9 times (HR = 2.9, *p* = 0.015, 95% CI = 1.23–6.88). Neither minor nor major adverse effects of low-dose MMI therapy were observed during the study period.

**Conclusions:**

In Graves' hyperthyroid patients with no or nonsevere ophthalmopathy who have completed a standard course of methimazole therapy without an adverse effect and have achieved an euthyroid status that is stably maintained with low-dose methimazole, a long-term continuation of the low-dose methimazole of 2.5–5 mg daily is effective and safe in the prevention of recurrent hyperthyroidism or maintenance of euthyroid status as long as the low-dose methimazole is continued. (TCTR20170705002).

## 1. Introduction

Treatment of Graves' hyperthyroidism (GH) includes medical therapy with a thionamide antithyroid drug (ATD) either propylthiouracil or more preferably methimazole (MMI), and ablative therapies with either radioactive iodine or near-total or total thyroidectomy [1–3]. Among them, ATD therapy is most commonly chosen as the first line of treatment [4–10]. A major problem of ATD therapy is recurrent hyperthyroidism (RH) that commonly occurs after discontinuation of ATD at a high rate of 35%–50% [11, 12]. A meta-analysis of 26 randomized controlled studies on the ATD regimens in adult patients with GH showed that the duration of ATD therapy with either a titration regimen or block and replacement regimen is a major factor associated with the development of RH after discontinuation of ATD, and the ATD therapy should be continued for 12–18 months, as a treatment duration of longer than 18 months does not increase the remission rate or decrease the recurrent rate after discontinuation of ATD [11]. To circumvent the problem of a high rate of RH after ATD discontinuation, long-term continuation of low-dose ATD to maintain euthyroid status or prevent RH is an alternative management commonly offered to patients with GH, particularly those who have RH and do not prefer the ablative therapy. However, this management is recommended by the American Thyroid Association [3] with low quality of supporting evidence obtained from only a few retrospective or nonrandomized control studies [13, 14]. To the best of our knowledge, there is no randomized controlled study primarily aimed at verifying the benefit of long-term continuation of ATD therapy. The present prospective randomized controlled study was, therefore conducted to examine whether long-term continuous low-dose ATD therapy beyond the recommended duration of ATD therapy would provide a benefit in the maintenance of euthyroid status or prevention of RH in GH patients who achieved euthyroid status with a standard course of ATD therapy that were eligible for discontinuation of ATD.

## 2. Patients and Methods

### 2.1. Prerandomization Recruitment of Eligible Patients

**Figure 1:**
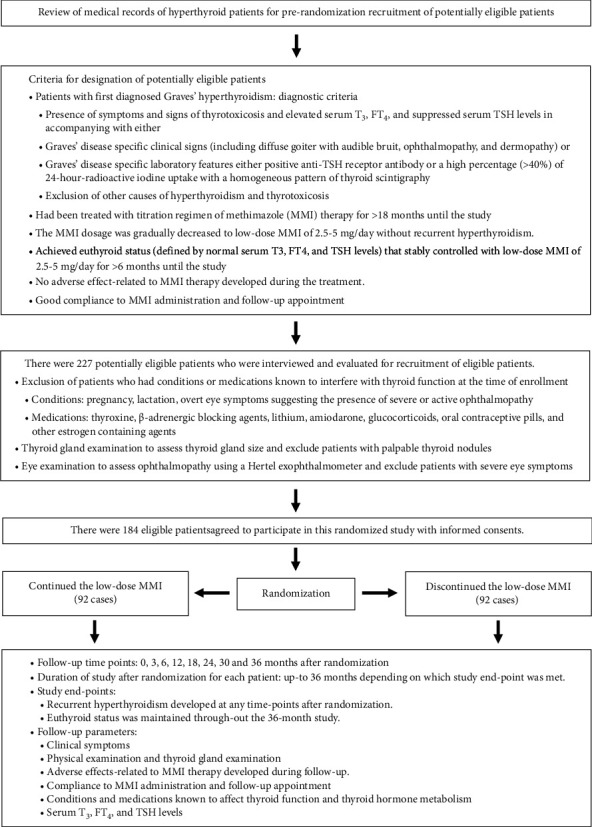
Prerandomization recruitment of eligible patients, randomization, and follow-up.

Medical records of patients with GH who were regularly treated and followed up at the endocrine clinic of our institute were reviewed for inclusion and exclusion criteria to designate potentially eligible patients for enrollment in this prospective randomized study, i.e., patients with first diagnosed GH who were being treated with the first course of a standard titration regimen of MMI therapy for 18 months or longer achieved a euthyroid status that was stably maintained with a low daily dosage of 2.5–5 mg/day of MMI for at least 6 months prior to the enrollment without adverse effects of MMI and recurrent hyperthyroidism developed during the treatment as shown in Figure 1. GH was diagnosed by the presence of symptoms and signs of thyrotoxicosis, elevated serum thyroid hormones with suppressed serum thyroid-stimulating hormone (TSH) levels in accompanying with Graves' disease specific characteristics either clinical signs (diffuse goiter with audible bruit, ophthalmopathy, and dermopathy) or laboratory features (positive serum anti-TSH receptor antibody (TRAb) or a high percentage (>40%) of 24-hour radioactive iodine uptake (24-hour RAIU) with a homogeneous pattern of thyroid scintigraphy) according to the American Thyroid Association (ATA)/American Association of Clinical Endocrinologists (AACE) 2011 and the ATA 2016 guidelines [1, 3]. In addition to using Graves' disease specific characteristics, the possibility of designating patients with other common causes of hyperthyroidism and thyrotoxicosis such as drug-induced hyperthyroidism and thyrotoxicosis, toxic multinodular goiter, toxic adenoma, and subacute thyroiditis [1, 3] was minimized by excluding patients who had the following conditions at first diagnosis of GH and during follow-up: medications known to cause hyperthyroidism and thyrotoxicosis, single or multiple thyroid nodules detected by palpation or thyroid scintigraphy, painful goiter, and euthyroidism or hypothyroidism rapidly developed within 8 weeks after the initiation of MMI therapy. The potentially eligible patients were evaluated by interview and general physical examination, including thyroid gland palpation and eye examination, by one investigator (R.L.), to determine patients who were fully eligible to participate in this study. Patients who had the following conditions before or at the time of enrollment were also excluded: pregnancy, lactation, overt eye symptoms (i.e., severe irritation, pain, diplopia, and visual impairment) suggesting the presence of severe or active ophthalmopathy that needed to be assessed and managed by ophthalmologists [3, 15], history of RH developed while decreasing MMI dosage, noncompliance to MMI administration and follow-up appointment, history of adverse effects related to MMI therapy and receiving medications known to interfere with thyroid function and thyroid hormone metabolism including [1, 3] thyroxine, *β*-adrenergic blocking agents, lithium, amiodarone, glucocorticoids, oral contraceptive pills, and other estrogen containing agents. The size of the thyroid gland was assessed clinically using the 1960 World Health Organization (WHO) palpation system and was classified into 4 groups, including no goiter (WHO grade 0, thyroid not palpable or if palpable not larger than normal), small goiter (WHO grade 1, goiter detectable only by palpation and not visible or visible when the neck is fully extended), moderate goiter (WHO grade 2, goiter visible when the neck is in its normal position and palpation not needed for diagnosis), and large goiter (WHO grade 3, goiter recognizable from a considerable distance) [16]. Graves' ophthalmopathy was mainly defined by the presence of exophthalmos with or without eyelid retraction. A Hertel exophthalmometer (Oculus, Germany) was used to measure the proptosis of eyeballs, which is the distance between the lateral orbital rim and the most anterior position of the cornea. In our ethnic population, exophthalmos was diagnosed when the proptosis was >16.5 mm in men and >16.1 mm in women [3, 17]. Of the 227 potentially eligible patients, there were 184 eligible patients who agreed to participate in this study and gave informed consent. Among them, 117 cases had GH diagnosed based on the presence of hyperthyroidism with one or more Graves' disease specific clinical signs. In 67 patients who had none of Graves' disease specific clinical signs, 55 cases had GH diagnosed by the presence of hyperthyroidism with positive TRAb, and 12 cases who were not tested for TRAb had GH diagnosed by the presence of hyperthyroidism with a high 24-hour RAIU and a homogeneous pattern of thyroid scintigraphy.

### 2.2. Randomization

184 eligible patients were randomly divided into 2 groups by using a computer-generated block of 4 randomization methods (nQuery Advisor 6.01). One group (92 cases) was assigned to discontinue MMI (DISCONT-MMI) and the other group (92 cases) was assigned to continue MMI (CONT-MMI) at the dosage that the patients had been taking before randomization, as shown in Figure 1. After randomization, the patients in both groups were evaluated at 3, 6, 12, 18, 24, and 36 months for clinical symptoms and signs of hyperthyroidism; adverse effects of MMI; thyroid function including serum total T_3_ (TT_3_), free T_4_ (FT_4_), and TSH levels; compliance to MMI administration assessed by pill-counting; compliance to follow-up; and conditions and medications that might interfere with thyroid function and thyroid hormone metabolism. Patients who had one or more of the following conditions after randomization were not included in the analysis: failure to take the MMI of more than 90% of the prescribed amount, failure to follow-up for longer than 6 months, and conditions or medications known to interfere with thyroid function. Euthyroid status was defined by the presence of serum TT_3_, FT_4_, and TSH levels within their normal ranges. RH was defined by the presence of elevated serum TT_3_ and/or FT_4_ in accompanying with suppressed serum TSH levels. The duration of the study for each patient was up to 36 months after randomization, depending on which study end-point was met. The end-point of the study for each patient was determined by the occurrence of RH at any follow-up time points or the remaining euthyroid status throughout the maximal study duration of 36 months. This study was approved by the Human Research Committee of our institute and by the Thai Clinical Trials Registry (TCTR) with a TCTR identification number TCTR20170705002.

### 2.3. Biochemical Analyses

All hormonal and other biochemical measurements were performed in a laboratory accredited by the International Organization for Standardization (ISO 15189) and were monitored by the Randox International Quality Assessment Scheme (RIQAS). Serum TT_3_, TT_4_, FT_4_, and TSH were measured by the electrochemiluminescent immunoassay (CLIA) method using an automated machine (Modular E170, USA). The normal ranges were 80.0–200.0 ng/dL (1.22–3.07 nmol/L) for serum TT_3_, 5.09–14.10 *μ*g/dL (65.6–181.5 nmol/L) for serum TT_4_, 0.93–1.94 ng/dL (12.0–25.0 pmol/L) for serum FT_4_, and 0.23–4.00 mIU/L for serum TSH levels. TRAb was measured by fast enzyme-linked immunosorbent assay using a porcine TSH receptor as an antigen (Euroimmun, Germany) with a positive cut-off level of >1 IU/L.

### 2.4. Statistical Analysis

Based on the results of a previous prospective non-randomized study by Morales et al. showing that RH after discontinuation of MMI therapy was 77.4% and during MMI therapy was 22.6% [14], a sample size of at least 45 cases per group was estimated to provide a significant difference in the rate of RH with an 80% power, a type 1 error of 0.05, and a type 2 error of 0.2. Results are expressed as percent or mean ± SD as appropriate. The Chi-square test and Student's *t*-test were used to compare the categorical and continuous data, respectively, between the two groups. The Kaplan-Meier survival analysis was used to compare the recurrent rate of Graves' hyperthyroidism between both groups, using the date of randomization as time 0 for the analysis. A Cox-proportional hazard model was used to examine the effects of factors associated with RH, including continuation of low-dose antithyroid drug therapy, age of onset of Graves' hyperthyroidism, sex, family history of hyperthyroidism, thyroid size, presence of ophthalmopathy or dermopathy, duration of antithyroid drug therapy, serum TT_3_, TT_4_, and serum TT_3_/TT_4_ ratio at the first diagnosis of Graves' hyperthyroidism. A *p* value of <0.05 was considered statistically significant. All statistical analyses were performed using the Statistical Package for the Social Sciences (SPSS) version 25.

## 3. Results

184 patients with Graves' hyperthyroidism who had been treated with the first course of MMI therapy for at least 1 year and achieved euthyroid status with low-dose MMI of 2.5–5 mg/day for at least 6 months were recruited for randomization. After randomization, there were 92 patients in the CONT-MMI group and 92 patients in the DISCONT-MMI group. Of the 55 patients who had GH diagnosed by TRAb results, 26 cases were in CONT-MMI and 29 cases were in DISCONT-MMI. Eleven patients, including 9 cases in CONT-MMI and 2 cases in DISCONT-MMI, were excluded from the analysis due to loss to follow-up (8 cases), noncompliance with MMI administration (2 cases), and pregnancy (1 case). At the end of the study, a total of 173 patients, including 83 cases in CONT-MMI and 90 cases in DISCONT-MMI, were eligible for the analysis. Among them, 25 cases in CONT-MMI and 27 cases in DISCONT-MMI had GH diagnosed by TRAb results.

Baseline patient characteristics at randomization of patients in the CONT-MMI and DISCONT-MMI groups are shown in Table 1. There was no significant difference in age, gender, prevalence of smoking, goiter, thyroid bruit, dermopathy, initial serum TT_3_, TT_4_ and TT_3_/TT_4_ ratio, and positive thyroid antibodies at first diagnosis between both groups. However, the DISCONT-MMI had a significantly higher prevalence of positive family history of hyperthyroidism (16.7% *vs.* 12.8%, *p* = 0.008) and a lower prevalence of ophthalmopathy (13.3% *vs.* 25.3%, *p* = 0.048) than those in the CONT-MMI. There was no difference in duration of MMI therapy prior to randomization, goiter size, and serum TT_3_, FT_4_, and TSH levels at randomization.

**Figure 2:**
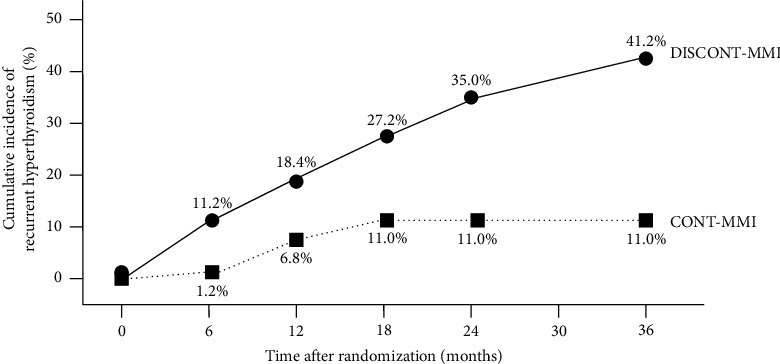
Cumulative rates of recurrent hyperthyroidism in Graves' hyperthyroid patients who were randomly assigned to continue (CONT-MMI) (83 cases) represented in a dotted line and to discontinue low-dose methimazole therapy (DISCONT-MMI) (90 cases) represented in a solid line.

**Figure 3:**
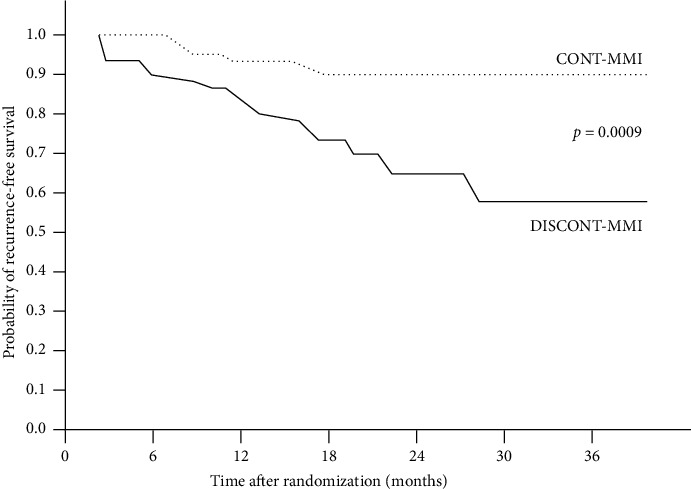
The Kaplan-Meier curve shows that 83 patients who were randomly assigned to continue low-dose methimazole therapy (CONT-MMI) represented in a dotted line had significantly higher recurrence-free survival than 90 patients who were randomly assigned to discontinue low-dose methimazole therapy (DISCONT-MMI) represented in a solid line (*p*=0.0009) with a Cox hazard ratio of 0.27 (95% CI = 0.12–0.62).

After randomization, there was no difference in follow-up duration between DISCONT-MMI and CONT-MMI (19.43 ± 13.9 *vs.* 20.17 ± 11.8 months, *p*=0.708). The cumulative rates of RH in CONT-MMI were significantly lower than those in DISCONT-MMI at every follow-up time point, and the significant difference was observed as early as within the first 6 months of the study (1.2% *vs.* 11.2%, 6.8% *vs.* 18.4%, 11.0% *vs.* 27.2%, 11.0% *vs.* 35.0%, and 11.0% *vs.* 41.2% at 6, 12, 18, 24, and 36 months, respectively) as shown in Table 2 and Figure 2. The Kaplan-Meier curve showed that patients who continued low-dose MMI after achieving euthyroid status (CONT-MMI) had significantly higher recurrence-free survival than those who discontinued MMI after achieving euthyroid status (DISCONT-MMI) (*p*=0.0009) with a Cox hazard ratio (HR) of 0.27 (95%CI = 0.12–0.62) as shown in Figure 3.

To determine the potential effect of long-term continuation of low-dose MMI therapy and other factors that have been previously shown to be associated with the risk of RH, Cox proportional hazard univariate analysis of all the 173 patients in both groups was performed and showed that there were 3 factors associated with the development of RH, including continuation of low-dose MMI therapy (HR = 0.27, *p*=0.002, 95% CI = 0.116–0.617), age onset of hyperthyroidism before 40 years (HR = 3.676, *p*=0.001, 95% CI = 1.657–8.157), and a positive family history of Graves' hyperthyroidism (HR = 2.466, *p*=0.032, 95% CI = 1.078–5.640) as shown in Table 3. The Cox proportional hazard multivariate analysis was subsequently performed and it showed that there were only 2 factors independently associated with the risk of RH, including continuation of low-dose MMI therapy, which decreased the risk of RH by 3.8 times (HR = 0.26, *p*=0.007, 95% CI = 0.10–0.70) and age onset of hyperthyroidism before 40 years, which increased the risk of RH by 2.9 times (HR = 2.9, *p*=0.015, 95% CI = 1.23–6.88) as shown in Table 4. Neither major and minor adverse effects of MMI nor worsening of ophthalmopathy were observed during the study period.

## 4. Discussion

The present 36-month prospective randomized controlled study primarily aimed to examine whether long-term continuation of low-dose MMI would provide a benefit in the maintenance of euthyroid status and prevention of RH compared to discontinuation of MMI in GH patients who accomplished an at least 18-month duration of ATD therapy and achieved euthyroid status that was stably maintained with low-dose MMI for at least 6 months that met the clinical criteria eligible for ATD discontinuation in accordance with the guidelines [1–3]. The results showed that patients who discontinued MMI (DISCONT-MMI), RH started to develop at as early as 3 months and thereafter the cumulative incidence of RH continuously increased to 11.2%, 18.4%, 27.2%, 35.0%, and 41.2% at 6, 12, 18, 24, and 36 months, respectively after discontinuation of the MMI, indicating that the rate of RH was very high and increases with time after ATD discontinuation despite the patients had met the eligible criteria for ATD discontinuation. The high rate of RH that increased with time after MMI discontinuation observed in this study was consistent with that reported in previous studies by Vichayanrat et al. (41% at 1 year) [18], Maugendre et al. (36% at 2 years) [19], Allannic et al. (38.2% at 2 years) [20], Garcia-Mayor et al. (54.1% at 2 years) [21], Morales et al. (77.42% at 5 years) [14], and Mazza et al. (43.5% at 5 years) [13]. In contrast, GH patients who continued low-dose MMI (CONT-MMI) had a rate of RH very much lower than that of the patients in DISCONT-MMI. The cumulative incidence of RH slowly increased to 11% at 18 months after randomization and stable thereafter. In addition, the lower cumulative incidence of RH was significantly observed as early as 3 months of the study and at every follow-up time point until the end of the 36-month study. Our findings indicated that in patients with GH who achieved euthyroid status with a standard course of MMI therapy and were eligible for MMI discontinuation, long-term continuation of low-dose MMI of 2.5–5 mg/day beyond the recommended 18-month duration of treatment provided a benefit in the maintenance of euthyroid status or prevention of RH, and the benefit persisted as long as the low-dose MMI therapy was still continued. The beneficial effect of long-term continuation of low-dose MMI in the prevention of RH was confirmed by the result of Cox proportional hazard multivariate analysis of 173 patients, including 83 cases in CONT-MMI and 90 cases in DISCONT-MMI, showing that continuation of low-dose MMI therapy was an independent factor associated with a decrease in the risk of RH by 3.8 times. The results of our prospective randomized controlled study agreed with those of previous retrospective or non-randomized controlled studies [13, 14, 22]. A retrospective observational uncontrolled study by Laurberg et al. in a cohort of 108 patients with GH and severe ophthalmopathy who were treated with low-dose thionamide, either MMI (101 cases) or PTU (7 cases) in combination with levothyroxine replacement, showed that 90% of the patients remained in euthyroid status throughout the median duration of therapy of 80 months [22]. A prospective nonrandomized study by Morales et al. in well-treated GH patients who remained in euthyroid status with low doses of 2.5–5 mg/day of carbimazole or MMI showed that patients who continued long-term low-dose maintenance of an ATD had a relapse rate (12 of 53 cases, 22.64%) significantly lower than of the patients who discontinued ATD (24 of 31 cases, 77.42%) during a 5-year follow-up. [14] A retrospective study by Mazza et al. of file data of 249 patients, 42 cases aged <35 years and 207 cases aged >35 years, with newly diagnosed GH were treated with a titration regimen of MMI therapy and achieved euthyroid status that was stably maintained with low-dose MMI of 2.5–5 mg/day showed that long-term continuation of treatment with low doses of MMI resulted in a significantly lower rate of RH compared to discontinuation of MMI therapy in patients over 35 years old (29 of 115 cases or 25.22% *vs.* 40 of 92 cases or 43.47%) but not in those under 35 years old (8 of 13 cases or 61.54% *vs.* 15 of 29 cases or 51.72%) [13]. Mazza et al. have also suggested that their findings should be confirmed by a prospective study. Our prospective randomized study shared partly similarity with the retrospective study of Mazza et al. in the patients' characteristics and study objectives that our and their patients were first diagnosed with Graves' disease were treated with MMI at the initial dose of 15–30 mg/day and the dosage was subsequently decreased to as low as 5 mg/day without RH before evaluating the benefit of long-term therapy with low-dose MMI in the prevention of recurrent hyperthyroidism or maintenance of euthyroid status. Whereas, most part of our study design were different from that of Mazza et al. study. The design of Mazza et al. study was totally retrospective in character that they reviewed file data of their patients who were observed in their clinic and compared the rate of recurrent hyperthyroidism in patients who discontinued with that of patients who continued the low-dose MMI (55 of 121 cases or 45.45% *vs.* 37 of 128 cases or 28.90%) by using the date of reduction of MMI dose to 5 mg/day as time 0 for survival analysis. Whereas, in our prospective study, we have reviewed the medical records of patients in our clinic to designated potentially eligible patients and carefully determined the eligible patients by excluding patients who had conditions that might confound the study outcome before and during the prospective follow-up after randomization. In addition, the date of randomization was used as time 0 for comparing the rate of RH between CONT-MMI and DISCONT-MMI. The higher rate of RH in patients younger than 35 years old observed in the study of Mazza et al. was consistent with that observed in the Cox proportional hazard multivariate analysis of our study showing that the age onset of hyperthyroidism before 40 years was an independent factor associated with an increased risk of RH by 2.9 times.

Long-term continuation of low-dose ATD therapy has also been shown to be equally or more effective in the prevention of RH or maintenance of euthyroid status with less hypothyroidism compared to not only ATD discontinuation but also RAI therapy [23, 24]. A retrospective study by Villagelin et al. in patients with RH after ATD discontinuation showed that after an average of 7 years duration of follow-up, long-term treatment with low-dose MMI of 2.5–7 mg daily in 114 cases was more effective in the maintenance of euthyroid status than combined RAI and L-thyroxine therapy in 102 cases, with a lower rate of subclinical and overt hypothyroidism, and the rate of euthyroid status was stable at almost 80% after 24 months of follow-up [24]. A randomized controlled study by Azizi et al. [23] aimed to compare the efficacy of continuous MMI therapy with that of RAI therapy in 104 patients who had recurrent hyperthyroidism showed that at 10 years of follow-up, continuous MMI therapy resulted in euthyroid status at a comparable rate as did the RAI, with less hypothyroidism, lower cost of treatment, and no serious complications.

In addition to the benefit in the maintenance of euthyroid status, long-term continuation of low-dose MMI therapy has also been shown to increase the remission rate after discontinuation of the drug in patients with GH [25–27]. Further study on the benefit in reduction of RH after discontinuation of long-term low-dose MMI therapy is under investigation in our patients.

The safety of long-term low-dose MMI therapy is also a major concern that makes many thyroidologists reluctant to continue the ATD after accomplishing a standard course of therapy. As did the previous studies [22–24, 28], our study found no adverse effects of long-term low-dose MMI therapy during the study period of at least 36 months, indicating that long-term low-dose MMI therapy is safe in GH patients who have accomplished a standard duration of ATD therapy without adverse effects. The absence of adverse effects observed in this as well as other studies can be explained by the results of a large retrospective study by Nakamura et al. [29] and a systematic review by Azizi and Malboosbaf [30] showing that the adverse effects of ATD, particularly MMI, occur commonly within the first 3 months and rarely in the later period of the therapy [29] as well as in patients with continuous use of MMI [24]. Therefore, it is less likely for patients who had no adverse effects during a standard course of ATD therapy to subsequently develop an adverse effect during long-term maintenance treatment with low-dose ATD.

The beneficial effect of long-term continuation of low-dose ATD in the maintenance of euthyroid status or prevention of RH might be explained by the direct inhibitory action on thyroid hormone synthesis or the immunosuppressive effects of ATD that are mediated primarily through decreasing thyroid specific autoimmunity or secondarily through ameliorating the hyperthyroid state that may subsequently restore the dysregulated immune system to normal [31].

Our study has some limitations. Firstly, the lack of TRAb results at the first diagnosis of GH in most (70%) of our patients might raise a major concern of selection bias in patient recruitment as the cause of hyperthyroidism in some of our patients might not be Graves' disease. However, the use of Graves' disease specific clinical signs, including diffuse goiter with bruit, ophthalmopathy, and dermopathy in this study should be acceptable for establishing the diagnosis of GH in accordance with the ATA/AACE 2011 and ATA 2016 guidelines that TRAb assay and RAIU tests may not be necessary in patients who have overt specific clinical features of Graves' disease [1, 3]. In addition, other common causes of hyperthyroidism or thyrotoxicosis such as drug-induced hyperthyroidism, toxic adenoma, toxic multinodular goiter, and thyroiditis were vigorously excluded by not enrolling patients who had a history of taking any drugs that were known to induce hyperthyroidism or thyrotoxicosis, single or multiple palpable thyroid nodules, and painful goiter. The comparable proportion of patients who had and had no TRAb results at the first diagnosis of GH in CONT-MMI (26 and 66 cases, respectively) and DISCONT-MMI (29 and 63 cases, respectively) at randomization indicated that there was no bias in the diagnosis of GH and prerandomization patient recruitment in patients who had not been assessed for TRAb. Secondly, as TRAb is a useful parameter in the prediction of remission and recurrence of GH [32, 33], the lack of data on TRAb levels at the time of randomization might lead to the possibility that there might be unequal TRAb levels and the number of patients with positive and negative TRAb that might result in a different rate of RH between patients in CONT-MMI and DISCONT-MMI. However, the randomized controlled trial in character of our study should be able to minimize this possibility. Thirdly, as this study recruited only patients who were being treated with the first course of MMI therapy and had no history of recurrent hyperthyroidism, there might be an argument that our findings might not be similarly observed in patients with a history of recurrent hyperthyroidism who are at higher risk of RH [34] and therefore are more potential candidates for long-term low-dose MMI therapy. A study in patients with GH by Sjölin et al. has shown that the rate of recurrent hyperthyroidism after the first course and second course of antithyroid drug therapy was very high at 54.7% and 70.6%, respectively [34]. Therefore, the benefit of long-term treatment with low-dose MMI in the maintenance of euthyroid status or prevention of recurrent hyperthyroidism observed in this study should be more or less applicable to patients with recurrent hyperthyroidism after the second or more courses of antithyroid drug therapy. Lastly, since our study recruited only GH patients who had no or nonsevere and inactive ophthalmopathy and excluded patients with severe and active ophthalmopathy, who have been reported to have a higher risk of RH compared to patients with nonsevere and inactive ophthalmopathy [35], there might be some debate about whether long-term treatment with low-dose MMI would be beneficial in preventing RH in patients with severe and active ophthalmopathy. The findings of our study's multivariate analyses, which revealed that nonsevere and inactive ophthalmopathy was not the independent factor associated with RH, indicated that the presence of nonsevere and inactive ophthalmopathy did not affect the outcome of long-term treatment with low-dose MMI, as determined by the rate of RH. In this study, there were few patients who had overt eye symptoms (such as severe irritation, pain, diplopia, and visual impairment) suggesting the presence of severe or active ophthalmopathy that needed to be assessed and managed by ophthalmologists at the time of prerandomization recruitment were excluded because they might need specific treatments for the ophthalmopathy with glucocorticoids, immunosuppressive agents, and orbital surgery as well as ablative therapy with either thyroidectomy or radioactive iodine for GH that were known to affect the study outcome after the randomization. More research is needed to determine whether long-term treatment with low-dose MMI would be beneficial in the prevention of RH in GH patients with severe and active ophthalmopathy.

## 5. Conclusions

The present prospective randomized controlled study has shown that long-term continuation with low-dose MMI of 2.5–5 mg/day beyond the recommended treatment duration of 18 months is safe and effective in the maintenance of euthyroid status or prevention of recurrent hyperthyroidism in Graves' hyperthyroid patients who have no or nonsevere ophthalmopathy, and the effectiveness persists as long as the MMI therapy is still continued. The results of our study, in addition to those of previous retrospective or nonrandomized controlled studies [13, 14, 22] do support and strengthen the ATA's recommendation [3] to offer a long-term continuation of low-dose ATD therapy for maintenance of euthyroid status or prevention of recurrent hyperthyroidism to patients who have recurrent hyperthyroidism and do not prefer ablative therapy.

## Figures and Tables

**Table 1:** Baseline clinical and biochemical characteristics of 173 Graves' hyperthyroid patients who were randomly assigned to discontinue and continue low-dose methimazole therapy.

	DISCONT-MMI (90 cases)	CONT-MMI (83 cases)	*p* value
Female/male (cases)	77/13	70/13	0.823
Age at first diagnosis of hyperthyroidism (years)	39.9 ± 10.9	42.2 ± 14.9	0.260
Positive family history of hyperthyroidism in first-degree-relatives (cases/cases, %)	18/50, 16.7%	6/47, 12.8%	0.008
Positive history of smoking (cases/cases, %)	2/50, 4.0%	3/48, 6.3%	0.169
Goiter size at randomization^∗^: Grade 0–1/grade 2/grade 3 (cases)	55/33/2	44/38/1	0.443
Thyroid bruit at randomization (cases/cases, %)	7/90, 7.8%	5/83, 6.0%	0.650
Ophthalmopathy at randomization^∗∗^ (cases/cases, %)	12/90, 13.3%	21/83, 25.3%	0.045
Dermopathy at randomization (cases/cases, %)	0/90, 0%	1/83	0.296
Positive antithyroglobulin antibody at diagnosis (cases/cases, %)	7/56, 12.5%	2/48, 4.2%	0.132
Positive antithyroid peroxidase antibody at diagnosis (cases/cases, %)	15/56	14/48	0.787
Serum TT_3_ at first diagnosis of hyperthyroidism (ng/dL)	454.9 ± 192.5	452.8 ± 189.8	0.945
Serum TT_4_ at first diagnosis of hyperthyroidism (*μ*g/dL)	20.1 ± 5.6	19.0 ± 5.4	0.226
Serum TT_3_/TT_4_ ratio at first diagnosis of hyperthyroidism	22.7 ± 8.2	24.6 ± 13.3	0.307
Serum TT_3_ (ng/dL) at randomization	108.6 ± 73.8	112.4 ± 62.5	0.716
Serum FT4 (ng/dL) at randomization	1.22 ± 0.24	1.26 ± 0.18	0.219
Serum TSH (mIU/L) at randomization	2.15 ± 1.47	2.26 ± 1.35	0.609
Duration of MMI therapy at randomization (months)	36.8 ± 20.5	31.6 ± 19.2	0.336

DISCONT-MMI and CONT-MMI denoted patients who were randomly assigned to discontinue and continue low-dose methimazole therapy, respectively. ^∗^Goiter size was assessed clinically by one investigator (R.L.) using the 1960 WHO palpation system [16]. ^∗∗^Ophthalmopathy was assessed by one investigator (R.L.) and patients with eye symptoms suggesting the presence of severe and active ophthalmopathy were not included.

**Table 2:** Cumulative rates of recurrent hyperthyroidism in 173 patients randomly assigned to discontinue and continue low-dose methimazole therapy.

Time after randomization (months)	Cumulative rates of recurrent hyperthyroidism after randomization (%)	
	DISCONT-MMI (90 cases)	CONT-MMI (83 cases)
6	11.2	1.2
12	18.4	6.8
18	27.2	11.0
24	35.0	11.0
36	41.2	11.0

DISCONT-MMI and CONT-MMI denoted patients who were randomly assigned to discontinue and continue low-dose methimazole therapy, respectively.

**Table 3:** Univariate analysis of factors associated with recurrent hyperthyroidism in 173 Graves' hyperthyroid patients who were eligible for analysis at the end of the study.

Factors	Hazard ratio	*p* value	95% CI
Continuation of low-dose methimazole	0.270	0.002	0.116–0.617
Age onset of hyperthyroidism before 40 years old	3.676	0.001	1.657–8.157
Family history of hyperthyroidism	2.466	0.032	1.078–5.640
Sex	1.289	0.635	0.453–3.670
History of smoking	0.043	0.612	0–73.578
Goiter size at randomization^∗^	0.676	0.082	0.318–1.436
Thyroid bruit at randomization	1.492	0.454	0.524–4.252
Ophthalmopathy at randomization^∗∗^	0.589	0.322	0.027–1.677
Dermopathy at randomization	0.049	0.705	0.00–292401
Serum TT_3_ at first diagnosis of hyperthyroidism	1.645	0.113	0.55–4.918
Serum TT_4_ at first diagnosis of hyperthyroidism	0.916	0.157	0.295–2.842
Serum TT_3_/TT_4_ at first diagnosis of hyperthyroidism	1.246	0.566	0.588–2.637
Antithyroglobulin antibody at first diagnosis of hyperthyroidism	0.043	0.374	0.00–43.686
Antimicrosomal antibody at first diagnosis of hyperthyroidism	1.343	0.530	0.535–3.371
Duration of methimazole therapy before randomization	0.988	0.257	0.968–1.009

^∗^Goiter size was assessed clinically using the 1960 WHO palpation system [16]. ^∗∗^Patients with eye symptoms suggesting the presence of severe and active ophthalmopathy were not included.

**Table 4:** Multivariate analyses of factors associated with recurrent hyperthyroidism in 173 Graves' hyperthyroid patients who were eligible for analyses at the end of the study.

Factors	Hazard ratio	*p* value	95% CI
Continuation of low-dose methimazole	0.26	0.007	0.10–0.70
Age onset of hyperthyroidism before 40 years old	2.9	0.015	1.23–6.88

## Data Availability

All the data obtained and analyzed during this study are included in this article.
